# Preoperative Peritoneal MRI: Usefulness to Highlight Potential Hidden Lesions for Complete Cytoreductive Surgery in Patients with Colorectal Cancer with Surgical History

**DOI:** 10.1245/s10434-025-18022-0

**Published:** 2025-08-06

**Authors:** Boris Cleret de Langavant, Amaniel Kefleyesus, Julien Peron, Olivier Glehen, Alexandre Galan, Nazim Benzerdjeb, Laurent Villeneuve, Vahan Kepenekian, Pascal Rousset, Rémi Grange

**Affiliations:** 1 Department of General Surgery and Surgical Oncology, CHU Lyon-Sud, Hospices Civils de Lyon, Pierre-Bénite, France; 2 CICLY, EMR 3738, Lyon 1 University, Lyon, France; 3 Department of Visceral Surgery, Lausanne University Hospital, and University of Lausanne, Lausanne, Switzerland; 4 Department of Medical Oncology, CHU Lyon Sud, Hospices Civils de Lyon, Pierre-Bénite, France; 5 Department of Radiology, CHU Lyon Sud, Hospices Civils de Lyon, Pierre-Bénite, France; 6 Department of Pathology, CHU Lyon Sud, Hospices Civils de Lyon, Pierre-Bénite, France; 7 Department of Clinical Research, CHU Lyon Sud, Hospices Civils de Lyon, Pierre-Bénite, France; 8 Department of Radiology, University Hospital of Saint-Etienne, Saint-Priest-en-Jarez, France

**Keywords:** Colorectal peritoneal metastases, MRI, Colorectal cancer, Cytoreductive surgery, Hyperthermic intraperitoneal chemotherapy

## Abstract

**Background:**

In patients with colorectal cancer and peritoneal metastases (CRC-PM), the completeness of cytoreductive surgery (CRS) is crucial. However, a history of moderate (Prior Surgical Score, PSS-2) or extensive (PSS-3) abdominal surgery may compromise the exploration, increasing the risk of undetected CRC-PM. This retrospective monocentric study investigated the value of preoperative peritoneal magnetic resonance imaging (MRI) in identifying potentially occult lesions in patients with PSS-2/3 CRC-PM scheduled for CRS.

**Patients and Methods:**

Consecutive patients with pathologically confirmed CRC-PM and PSS-2/3, selected for radical treatment, were included. All underwent preoperative peritoneal MRI ≤ 7 days before CRS, between January 2015 and December 2020. MRI, surgical, and pathological reports were reviewed focusing on seven anatomical sites of interest (perihepatic, pelvic, retroperitoneum, abdominal wall, anastomosis, inguinal canal, and cardiophrenic space).

**Results:**

Overall, 248 patients were included; 242 (97.6%) underwent complete CRS (CC-0). Among them, 212 (85.5%) were PSS-2 and 36 (14.5%) PSS-3. The sensitivity, specificity, and accuracy of MRI in detecting lesions were, respectively, 65%, 91%, and 82% (perihepatic region); 53%, 81%, and 63% (pelvis); 41%, 91%, and 69% (retroperitoneum); 46%, 91%, and 79% (abdominal wall); and 44%, 98%, and 74% (anastomotic sites). In the inguinal canal and cardiophrenic space, preoperative MRI led to ten resections in ten patients, with neoplastic cells detected in eight cases (80%).

**Conclusions:**

Preoperative peritoneal MRI demonstrated good specificity and a promising negative predictive value (NPV) but modest sensitivity in detecting lesions across seven anatomically challenging regions. Further studies are warranted to better define its added value over standard preoperative imaging protocols.

Colorectal cancer (CRC) is the third leading cause of cancer-related mortality worldwide.^1^ Up to 15% of these patients develop peritoneal metastases (PM), half being diagnosed metachronously.^2,3^ Globally, in nearly 50% of cases, PM remains confined to the peritoneal cavity.^4,5^ Among metastatic sites, the peritoneum is associated with the poorest prognosis, with a reported median overall survival of 16.3 months following palliative chemotherapy.^6^ However, in selected patients, complete cytoreductive surgery (CRS), potentially combined with hyperthermic intraperitoneal chemotherapy (HIPEC), can significantly extend that median survival to 41.6 months.^4,7,8^ The completeness of CRS, as assessed by the Completeness of Cytoreduction score (CC-score), remains the primary prognostic factor. Accurate preoperative evaluation of PM localizations and meticulous patient selection are therefore crucial. To date, surgical exploration remains the standard for mapping PM extent.^9,10^ However, prior abdominal surgeries—whether for benign conditions, primary tumor resection, or previous CRS—often led to adhesions, potentially limiting the quality of peritoneal assessment. Such surgical history is categorized using the Prior Surgical Score (PSS) as moderate (PSS-2) or extensive (PSS-3).^11^ Achieving a truly complete CRS in such cases requires a thorough peritoneal exploration, including previously dissected regions. The higher the PSS is, the more extensive the required dissection and the higher the risk of uncomplete CRS and potentially additional morbidity will be. A retrospective study, including 407 CRC-PM patients treated with CRS-HIPEC, showed that PSS-2 and PSS-3 patients had only 68.1% and 48.6% of complete resection (CC-0/1), respectively, with PSS being an independent factor for poor prognosis.^11^ A total of seven anatomical regions—perihepatic region, pelvis, retroperitoneum, abdominal wall, anastomosis, inguinal canal, and cardiophrenic space—are particularly prone to harboring occult PM in patients with prior surgery. Moreover, several of these regions remain imperfectly explored by staging laparoscopy. These areas are referred to as hidden regions.

Preoperative imaging plays a pivotal role in identifying and localizing PM to ensure complete CRS. Computed tomography (CT) scan is the preferred imaging modality to assess CRC distant metastasis.^12–15^ However, despite its excellent spatial resolution, CT scan is limited by low soft-tissue contrast, reducing its ability to distinguish small PM.^16,17^ In contrast, diffusion-weighted sequences combined with contrast-enhanced magnetic resonance imaging (MRI) showed promising results in assessing PM extension, especially in patients with mucinous PM.^5,18^ A recent meta-analysis has demonstrated the superiority of MRI over CT scan in this setting.^7,18,19^

Thus, improving the preoperative determination of PM location in patients with PSS-2/3 CRC-PM may contribute to optimizing surgical strategy, reducing the risk of missing hidden lesions, and ultimately increasing the chances of achieving a complete CRS. We hypothesized that, beyond assessing overall resectability, early preoperative peritoneal MRI may provide specific value in evaluating these seven anatomically challenging areas in patients with prior surgical history.

This retrospective monocentric study aimed at evaluating the performance of preoperative peritoneal MRI in that particular setting of patients with PSS-2/3 CRC-PM scheduled for CRS-HIPEC, considered at elevated risk of hidden PM and of severe morbidity induced by iterative dissections.

## Patients and Methods

This study was performed according to the ethical standards of the World Medical Association Declaration of Helsinki and approved by the Institutional Review Board of the Hospices Civils de Lyon. The informed consent requirement was waived by the ethics committees on the basis of the nature of this retrospective study, in which patient data were kept confidential and anonymized.

### Population

This retrospective, monocentric study included patients treated between 1 January 2015 and 1 December 2020. All cases were reviewed during a multidisciplinary team meeting, involving surgical oncologists, medical oncologists, pathologists, and radiologists, experts in peritoneal surface malignancies. Inclusion criteria were consecutive patients with pathologically confirmed PM from CRC and who had undergone prior surgery (i.e., PSS-2 or PSS-3) and were selected for radical treatment by CRS-HIPEC (considered resectable). Pathological confirmation of PM was obtained during the initial surgery, a staging laparoscopy, or a previous CRS. A preoperative peritoneal MRI had to be performed ≤ 7 days before CRS-HIPEC, which had to be complete (CC-score of 0 or 1). Exclusion criteria included appendicular neoplasia, unavailable MRI, no previous surgery, un-vailable surgical and/or pathological reports, and incomplete CRS (CC-2/3).

This study focused on the contribution of located in anatomically challenging regions. The primary objective was to reduce the risk of missed lesions in hidden regions, thus enabling an improved surgical strategy. In this setting, the overall resectability of PM had already been determined during the initial preoperative workup, which served as the basis for selecting patients for radical-intent treatment with CRS. That previous preoperative evaluation focused on minimizing the risk of unresectability, particularly in small bowel, bladder trigona, or hepatic pedicle involvement. Consequently, these regions were not considered as areas of interest in the present study. Baseline population characteristics included standard clinical, pathological, molecular, and treatment-related data. PM were defined as synchronous, diagnosed at the time of the initial CRC diagnosis or within 6 months thereafter. The PSS was classified between PSS-2 and PSS-3 according to previous surgery (PSS-2: exploratory laparotomy with dissection in two to five regions and PSS-3: extensive prior cytoreduction in more than five regions).^20^

### Preoperative MRI protocols

The preoperative imaging protocol included a peritoneal MRI, performed within 7 days prior to surgery. Examinations were performed using a 3T MR unit (Ingenia, Philips Medical Systems, Best, the Netherlands) equipped with an external phased-array surface coil. Images were acquired across the upper and lower abdomen to cover the entire abdominopelvic region. To reduce peristaltic motion artefacts, patients received an intravenous 1-mg dose (1 mg/mL) of Glucagon (Novo Nordisk, Copenhagen, Denmark) prior to the examination. MRI protocol included T2-weighted (slice thickness was 5 mm), fat suppressed T1-weighted (slice thickness was 4 mm), and diffusion-weighted whole-body imaging with background body signal suppression (DWIBS) (*b* values 0 and 1000 s/mm^2^; slice thickness was 5 mm) sequences in the axial plane, followed by fat suppressed T1-weighted sequences (slice thickness was 4 mm) in the axial and coronal planes, obtained 5 min after intravenous administration of gadobenate dimeglumine at a dose of 0.2 mL/kg (Multihance) (Bracco, Milano, Italy).

All peritoneal MRI were analyzed by a single specialized peritoneal radiologist (PR). The structured report integrated the description of peritoneal lesions and the evaluation of unresectable lesions or surgically complex lesions, as well as lesions at risk of being missed intraoperatively (hidden lesions). Radiological reporting was standardized using the PeRitOneal Malignancy Stage Evaluation (PROMISE) software.^21–23^ In this study, MRI reports were reviewed to identify all positive descriptions of PM in seven predefined areas of interest (perihepatic region, pelvis, retroperitoneum, abdominal wall digestive anastomosis, inguinal canal, and cardiophrenic space). The perihepatic region included the right subphrenic and subhepatic spaces. The retroperitoneum was defined as the space posterior to the parietal peritoneum and anterior to the posterior abdominal wall, excluding the psoas muscles and quadratus lumborum. The abdominal wall included partially or entirely located lesions within the rectus abdominis, internal or external oblique, and transverse muscles, with specific attention to laparotomy scars, epigastric vessels, and former trocar holes. The surgical pelvic region corresponded to the previously described region.^24^ Anastomoses referred to any recurrence in contact with a digestive anastomosis. The inguinal canal and cardiophrenic space were also assessed, as they are not systematically explored during CRS. The cardiophrenic region was defined as the fat‐filled space between the mediastinum, heart base, lung, diaphragm, and chest wall.

### Surgery

The surgical objective was the complete resection of all macroscopic peritoneal metastases, combining peritonectomies and organ resections as required. A complete surgical exploration of the abdominal cavity was performed, including previously dissected areas and prior anastomosis sites. On the basis of preoperative MRI findings, a targeted inspection and palpation of these regions (especially at anastomotic sites) was performed. Following inspection of the tagged regions, it was ultimately the surgeon’s decision whether to perform resections. In regions that are particularly difficult to access and not systematically explored—such as the cardiophrenic space and inguinal canal—surgical exploration was performed only in the presence of a positive MRI finding.

PM extension was quantified according to the Peritoneal Carcinomatosis Index (PCI). The CC-score was defined as follow: CC-0, no macroscopic residual tumor; CC-1, residual tumors < 2.5 mm; CC-2, residual tumors between 3.5and 25 mm; and CC-3, residual tumors > 25 mm. HIPEC was performed as previously described.^25^ Postoperative complications were reported and graded according to the Clavien–Dindo Classification.^26^ Complications graded ≥ III were considered major. Postoperative complications and mortality were defined as events occurring within 90 days after surgery or prior to hospital discharge. Length of stay in intensive care unit (ICU) and in hospital were recorded.

### Pathologic Analysis

All surgical specimens were anatomically identified and analyzed by an expert pathologist in peritoneal surface malignancies. The presence or absence of residual disease was systematically recorded.

### Statistical Analysis

Patient and disease characteristics were described using both categorical and continuous variables. Categorical variables were expressed as counts and percentages, while continuous variables were reported as medians with corresponding interquartile range. All MRI, surgical, and pathology reports in the seven areas of interest were reviewed.

For the five regions routinely explored during CRS (perihepatic region, pelvis, retroperitoneum, abdominal wall, and anastomosis), the diagnosis performance of preoperative peritoneal MRI was assessed by calculating sensitivity (Se), specificity (Sp), positive predictive value (PPV), and negative predictive value (NPV). This evaluation was based on surgical and pathological reports. A region was considered a true positive if the MRI described a lesion subsequently confirmed by pathology. A false negative was defined as a lesion not detected by MRI but discovered and confirmed during surgery and pathological examination. Conversely, if the pathological analysis revealed PM in a resected specimen, it was likewise considered as false negative. The number of affected regions of interest per patient was classified on the basis of pathological outcomes. If a region was suspected of invasion on MRI but was ultimately deemed unaffected during surgical exploration and not resected, it was considered as negative (false-positive of MRI).

For the inguinal canal and cardiophrenic space, which are not systematically explored during CRS, a qualitative analysis was conducted. The number of MRI-suspected lesions in these regions and the proportion of pathologically invaded ones were reported. MRI performance in these two regions, as well as in the five others, could not be assessed because surgical dissection was performed only when MRI findings were positive. Statistical analyses were performed using R software (4.2.2 version).

## Results

### Patient Cohort

**Fig. 1:**
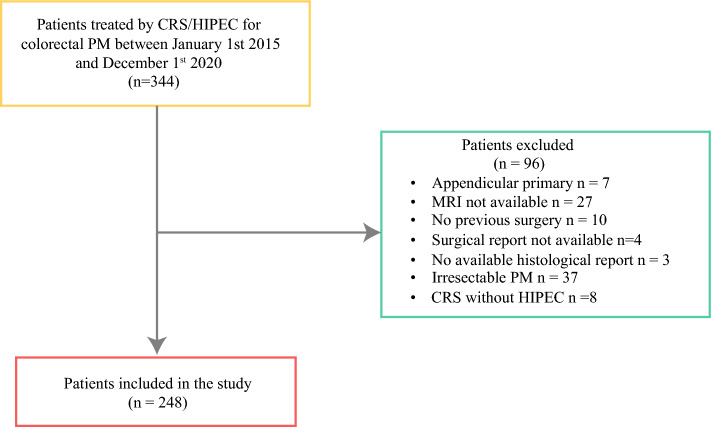
Flowchart of the study population (*n* = 248); *CRS* cytoreductive surgery, *HIPEC* hyperthermic intraperitoneal chemotherapy, *MRI* magnetic resonance imaging, *PM* peritoneal metastasis

The study flowchart is detailed in Fig. 1, showing that 248 (127 male, 51.2%) out of 331 patients with CRC-PM were included. The study population is described in Table 1. The median age was 61 [53–68] years; 67 (27.0%) patients had right-sided colon cancer, while 44 (17.7%) had left-sided colon cancer. Regarding previous surgery, 212 (85.5%) patients were classified as PSS-2, and 36 (14.5%) patients as PSS-3. The PM were metachronous in 161 (64.9%) patients and synchronous in 87 (35.1%) patients. The primary tumors were treated in emergency for occlusion in 27 (10.9%) patients, were pT4 in 110 (44.4%) patients, and pN2 in 78 (31.5%) patients (Table 2).

**Table 1:** Baseline characteristics of the study population

Baseline characteristics	*N* = 248
Sex (male), *n* (%)	127 (51.2)
Age (years)^a^	61 [53–68]
BMI (kg/m^2^)^a^	24.5 [21.1–27.9]
*pT stage of primary, n (%)*	
pT1	0 (0)
pT2	8 (3.2)
pT3	97 (39.1)
pT4	110 (44.4)
Unknown	33(13.3)
*pN stage of primary, n (%)*	
pN0	65 (26.2)
pN1	63 (25.4)
pN2	78 (31.5)
Unknown	42 (16.9)
Pathology, *n* (%)	
Intestinal-type adenocarcinoma	186 (75.0)
Mucinous adenocarcinoma	53 (21.4)
Signet ring cell	2 (0.8)
Other	7 (2.8)
*Discovery of primary, n (%)*	
Occlusion	27 (10.9)
Perforation	3 (1.2)
Systemic neoadjuvant chemotherapy, *n* (%)	234 (94.4)
Number of cycles^a^	5 [4–7]
*Mutations, n (%)*	
*KRAS mutation*	
Yes	83 (33.5)
No	98 (39.5)
Unknown	67 (27.0)
*BRAF mutation*	
Yes	20 (8.1)
No	161 (64.9)
Unknown	67 (27.0)
MMR status	
MSI	12 (4.8)
MSS	154 (62.1)
Unknown	82 (33.1)
*Status of PM, n (%)*	
Metachronous	161 (64.9)
Synchronous	87 (35.1)
Previous CRS/HIPEC, *n* (%)	7 (2.8)
*Prior Surgical Score, n (%)*	
PSS-2	212 (85.5)
PSS-3	36 (14.5)
*Location of primary, n (%)*	
Right colon	67 (27.0)
Transverse colon	15 (6.0)
Left colon	44 (17.7)
Sigmoid colon	90 (36.3)
Rectum	13 (5.2)
Unknown	19 (7.8)
*Median delay between PM diagnosis and CRS/HIPEC (days)*^*a*^	
PSS-2	156 [121–218]
PSS-3	182 [141–747]

^a^Values expressed as median [IQR]

*ASA* American Society of Anesthesiologists, *BMI* body mass index, *IQR* interquartile range, *MMR* mismatch repair, *MSI* microsatellite instability, *MSS* microsatellite stability, *PM* peritoneal metastasis, *pT* pathological tumor stage, *pN* pathological nodal stage

**Table 2:** Surgical data of the study population (N = 248)

Surgical characteristics	*N* = 248
*Completeness of cytoreduction, n (%)*	
CC-0	242 (97.6)
CC-1	6 (2.4)
Length of stay in ICU (days)^a^	2 [1–3]
Length of stay (days)^a^	15 [11–22]
Re-intervention, *n* (%)	36 (14.5)
Surgical PCI^a^	6 [3–10]
Surgical PCI in PSS-2 group^a^	5 [3–10]
Surgical PCI in PSS-3 group^a^	6 [3–10]
*Chemotherapy for HIPEC, n (%)*	
Mitomycin	122 (49.2)
Oxaliplatin	78 (31.4)
Cisplatin	48 (19.4)
Operation time (min)^a^	300 [245–390]
Follow-up (months)^a^	18.8 [10.0–33.5]
Mortality during follow-up, *n* (%)	48 (19.4)
90-days postoperative mortality, *n* (%)	6 (2.4)
*Type de complications, n (%)*	
Hematologic	42 (16.9)
Cardiovascular	21 (8.5)
Pulmonary	41 (16.5)
Gastrointestinal	36 (14.5)
Urological	19 (7.7)
Major complications (Clavien ≥ III), *n* (%)	129 (52.0)
Adjuvant chemotherapy,* n* (%)	180 (72.6)

^a^Values expressed as median[IQR]

*CC* completeness of cytoreduction, *HIPEC* hyperthermic intraperitoneal chemotherapy, *ICU* intensive care unit, *IC* interval confidence, *IQR* interquartile range, *PCI* peritoneal carcinomatosis index

### Preoperative MRI Performance

**Fig. 2:**
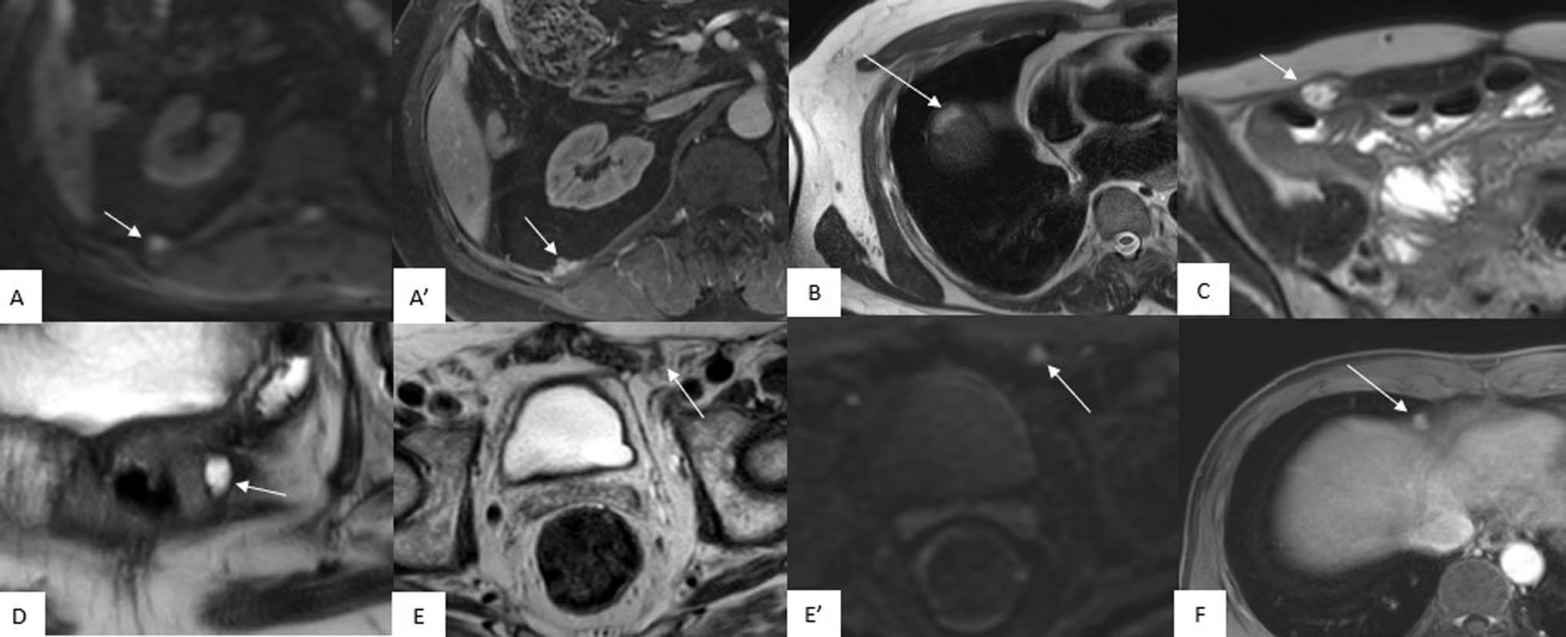
Preoperative MRI slides illustrating its usefulness for areas of interest: **AA′** retroperitoneum; **B** perihepatic region; **C** abdominal wall; **D** anastomosis; **E** and **E′** inguinal canal; **F** cardiophrenic space; all these lesions were detected by the radiologist, resected, and histopathological analysis confirmed tumoral involvement; **AA′** 56-year-old patient with previous right colon adenocarcinoma and CRS/HIPEC, axial diffusion-weighted (**A**) and axial post contrast T1-weighted with fat suppression (**A′**) show a right nodular lesion in the retroperitoneal in the deep space of right colectomy, behind the right kidney; **B** 47-year-old patient with a history of mucinous colon adenocarcinoma and CRS/HIPEC, axial T2-weighted image shows a mucinous recurrence in a previous area of peritonectomy and glisonnectomy with an 18-mm hyperintense nodule high located in the right inter-hepatic-diaphragmatic space; **C** 76-year-old patient with mucinous right colic neoplasia with synchronous peritoneal metastases, axial T2-weighted image shows an abdominal wall lesion with 16-mm hyperintense nodule on a trocar trajectory of the right iliac fossa (arrow); **D** 54-year-old patient with a history of mucinous sigmoid cancer, axial T2-weighted image shows subperitonised mucinous recurrence with a hyperintense nodule in contact with the left side of colorectal anastomosis (arrow); **EE′** 73-year-old patient with a history of sigmoid adenocarcinoma, axial T2-weighted (**E**) and axial diffusion-weighted (**E′**) images show a peritoneal nodule within the left inguinal region (arrows); **F** 47-year-old patient with a history of colon adenocarcinoma, axial post contrast T1-weighted with fat suppression image show a 10-mm round lymph node in the right cardiophrenic space (arrow)

Among the five regions of interest (perihepatic, pelvis, retroperitoneum, abdominal wall, and anastomosis), preoperative MRI identified suspicious lesions that were subsequently surgically resected in 89 (35.9%), 158 (63.7%), 110 (44.4%), 66 (26.6%), and 110 (44.4%) patients, respectively. In 45 (18.1%) patients, preoperative MRI was negative for all five target regions interest. Among them, the median surgical PCI was 4 [2–6]. Notably, in 24 (54.5%) patients, the indication was a systematic second look to treat potentially radiologically occult PM (Table 3). Representative MRI images are described in Fig. 2.

**Table 3:** Preoperative MRI, surgical and histological reports according regions of interest

Variables	*N* = 248
*Preoperative MRI, n (%)*	
No PM	44 (17.7)
In PSS-2 group	38 (86.4)
In PSS-3group	6 (13.6)
"Second-look" surgery	24 (54.5)
Surgical PCI^a^	4 [2–6]
*No PM in regions of interest*	45 (18.1)
In PSS-2 group	39 (86.7)
In PSS-3 group	6 (13.3)
*Positive MRI according region of interest*	
Perihepatic	73 (29.4)
Pelvis	100 (40.3)
Retroperitoneum	59 (23.8)
Abdominal wall	46 (18.6)
Anastomosis	52 (20.1)
*Surgical resection of region of interest, n (%)*	
No resection in regions of interest	9 (3.6)
*Resection according to the region of interest*	
Perihepatic	89 (35.9)
Pelvis	158 (63.7)
Retroperitoneum	110 (44.4)
Abdominal wall	66 (26.6)
Anastomosis	110 (44.4)
*Pathology, n (%)*	
No metastasis in all resected specimen	68 (27.4)
No metastasis in regions of interest	84 (33.9)
*Positive pathology according to the region of interest*	
Perihepatic	60 (67.4)
Pelvis	94 (59.5)
Retroperitoneum	35 (31.8)
Abdominal wall	23 (34.8)
Anastomosis	73 (66.4)

^a^Values expressed as median[IQR]

*MRI* magnetic resonance imaging, *PM* peritoneal metastases

### Surgery

Median surgical PCI was 6 [3–10]. The median duration of procedure was 300 [245–390] min. CRS was considered CC-0 and CC-1 in 242 (97.6%) and 6 (2.4%) patients, respectively. Major complications occurred in 129 (52.0%) patients. The median length of hospital stay was 15 [11–22] days and postoperative mortality was reported for 6 (2.4%) patients. Systemic neoadjuvant chemotherapy was administrated in 234 (94%) patients with a median of 5 [4–7] cycles, while 180 (75.6%) patients benefited from adjuvant chemotherapy (Table 2).

### Pathology

The perihepatic, pelvis, retroperitoneum, abdominal wall, and anastomosis regions of interest were pathologically positive in 60/89 (67.4%), 94/158 (59.5%), 35/110 (31.8%), 23/66 (34.8%), and 73/110 (66.4%) patients, respectively. In 84 (33.9%) patients, no specimen was positive for all five regions of interest, and in 68 (27.4%) patients, all surgical specimens were negative.

### Agreement between Preoperative MRI and Surgery

Overall Se, Sp, PPV, NPV, and accuracy of preoperative MRI were 65%, 91%, 80%, 79%, and 82% for perihepatic region, respectively; 53%, 81%, 83%, 49%, and 63% for the pelvis, respectively; 41%, 91%, 78%, 66%, and 69% for retroperitoneum, respectively; 46%, 91%, 66%, 82%, and 79% for abdominal wall, respectively; and 44%, 98%, 94%, 69%, and 74% for anastomosis, respectively (Table 4a).

**Table 4:** Diagnostic performances of MRI compared with surgery (n = 248)

Regions	*N*	Se	95% CI		Sp	95% CI		PPV	NPV	Accuracy
			Lower	upper		Lower	upper			
(A) Regions with a difficult and complex surgical dissection in the study population (n = 248)										
Perihepatic	73	65%	51%	77%	91%	83%	95%	80%	79%	82%
Pelvis	100	53%	45%	61%	81%	71%	89%	83%	49%	63%
Retroperitoneum	59	41%	32%	51%	91%	84%	95%	78%	66%	69%
Abdominal wall	46	46%	34%	59%	91%	86%	95%	66%	82%	79%
Anastomosis	52	44%	35%	54%	98%	94%	99%	94%	69%	74%
(B) Regions with a difficult and complex surgical dissection, in patients with previous moderate dissection (PSS-2) (n = 212)										
Perihepatic	61	64 %	52 %	74 %	90 %	84 %	94 %	77 %	82 %	81 %
Pelvis	85	52 %	43 %	61 %	81 %	70 %	89 %	82 %	50 %	63 %
Retroperitoneum	48	38 %	29 %	49 %	90 %	83%	95 %	75 %	65 %	67 %
Abdominal wall	35	40 %	27 %	55 %	91 %	86 %	95 %	60 %	83 %	79 %
Anastomosis	43	44 %	33 %	54 %	98 %	93 %	99 %	93 %	69 %	74 %
(C) Regions with a difficult and complex surgical dissection, in patients with previous extensive dissection (PSS-3) (n = 36)										
Perihepatic	12	73%	45%	92%	95%	76%	100%	92%	83%	86%
Pelvis	15	54%	33%	74%	83%	52%	98%	87%	48%	64%
Retroperitoneum	11	63%	35%	85%	95%	75%	100%	91%	76%	81%
Abdominal wall	11	64%	35%	87%	91%	71%	99%	82%	80%	81%
Anastomosis	9	50%	26%	74%	100%	82%	100%	100%	67%	75%

*CI* confidence interval, *PSS* Prior Surgical Score, *PPV* positive predictive value, *NPV* negative predictive value, *N* number of patients with confirmed lesions by specific region described during surgery (gold standard), *Se* sensitivity, *Sp* specificity, *accuracy* proportion of all patients with MRI identified peritoneal metastasis confirmed surgically

3.6 Impact of the Prior Surgical Score

Among PSS-2 patients, the overall Se, Sp, PPV, NPV, and accuracy of preoperative MRI were 64%, 90%, 77%, 82%, and 81% for perihepatic region, respectively; 52%, 81%, 82%, 50%, and 63% for the pelvis, respectively; 38%, 90%, 75%, 65%, and 67% for retroperitoneum, respectively; 40%, 91%, 60%, 83%, and 79% for abdominal wall, respectively; and 44%, 98%, 93%, 69%, and 74% for anastomosis, respectively (Table 4B).

Among PSS-3 patients, the overall Se, Sp, PPV, NPV, and accuracy of preoperative MRI were 73%, 95%, 92%, 83%, and 86% for perihepatic region, respectively; 54%, 83%, 87%, 48%, and 64% for the pelvis, respectively; 63%, 95%, 91%, 76%, and 81% for retroperitoneum, respectively; 64%, 91%, 82%, 80%, and 91% for abdominal wall, respectively; and 50%, 100%, 100%, 67%, and 75% for anastomosis, respectively (Table 4C).

### Agreement between Preoperative MRI and Pathology

The overall Se, Sp, PPV, NPV, and accuracy of preoperative MRI were 61%, 74%, 82%, 49%, and 66% for perihepatic region, respectively; 73%, 50%, 63%, 61%, and 62% for the pelvis, respectively; 53%, 89%, 67%, 82%, and 79% for retroperitoneum, respectively; 67%, 67%, 52%, 79%, and 67% for abdominal wall, respectively; and 53%, 69%, 76%, 43%, and 58% for anastomosis, respectively.

Impact of Prior Surgical Score among PSS-2 patients for Se, Sp, PPV, NPV, and accuracy of preoperative MRI were 56%, 74%, 79%, 49%, and 63% for perihepatic region, respectively; 61%, 61%, 75%, 45%, and 61% for the pelvis, respectively; 55%, 87%, 60%, 84%, and 79% for retroperitoneum, respectively; 62%, 74%, 59%, 76%, and 69% for abdominal wall, respectively; and 54%, 71%, 76%, 48%, and 61% for anastomosis, respectively (Table 5A).

**Table 5:** Diagnostic performances of MRI compared with pathology (n = 248)

Regions	*N*	Se	95% CI		Sp	95% CI		PPV	NPV	Accuracy
			Lower	upper		Lower	upper			
(A) regions with a difficult and complex surgical dissection in the study population (n = 248)										
Perihepatic	46	61%	42%	77%	74%	45%	92%	82%	49%	66%
Pelvis	81	73%	62%	82%	50%	38%	62%	63%	61%	62%
Retroperitoneum	12	53%	27%	79%	89%	73%	97%	67%	82%	79%
Abdominal wall	30	67%	51%	80%	67%	54%	78%	52%	79%	67%
Anastomosis	51	53%	41%	64%	69%	52%	83%	76%	43%	58%
(B) Regions with a difficult and complex surgical dissection, in patients with previous moderate dissection (PSS-2) (n = 212)										
Perihepatic	34	56%	41%	71%	74%	54%	89%	79 %	49%	63%
Pelvis	68	61%	50%	72%	61%	44%	75%	75%	45%	61%
Retroperitoneum	10	55%	23%	83%	87%	70%	96%	60%	84%	79%
Abdominal wall	22	62%	38%	82%	74%	56%	87%	59%	76%	69%
Anastomosis	42	54%	41%	67%	71%	54%	85%	76%	48%	61%
(C) Regions with a difficult and complex surgical dissection, in patients with previous extensive dissection (PSS-3) (n = 36)										
Perihepatic	12	75%	43%	95%	75%	19%	99%	90%	50%	75%
Pelvis	13	73%	39%	94%	64%	35%	87%	62%	75%	68%
Retroperitoneum	2	50%	7%	93%	100%	40%	100%	100%	67%	75%
Abdominal wall	8	100%	16%	100%	46%	17%	77%	25%	100%	54%
Anastomosis	9	50%	23%	77%	50%	7%	93%	78%	22%	50%

*CI* confidence interval, *PSS* Prior Surgical Score, *N* number of patients with confirmed lesions by specific region described at pathology (gold standard), *Se* sensitivity, *Sp* specificity, *Inf*. Inferior, *accuracy* proportion of all patients with MRI identified peritoneal metastasis confirmed surgically

Among PSS-3 patients, the Se, Sp, PPV, NPV and accuracy of preoperative MRI were 75%, 75%, 90%, 50%, and 75% for perihepatic region, respectively; 73%, 64%, 62%, 75%, and 68% for the pelvis, respectively; 50%, 100%, 100%, 67%, and 75% for retroperitoneum, respectively; 100%, 46%, 25%, 100%, and 54% for abdominal wall, respectively; and 50%, 50%, 78%, 22%, and 50% for anastomosis, respectively (Table 5B).

### MRI Impact for Regions Out of the Common Scope of Surgical Exploration

In PSS-2 patients, MRI identified lesions in the cardiophrenic space in two cases, resulting in two resections, both of which were pathologically positive. In the pelvis, MRI detected lesions in the inguinal canal in eight patients, resulting in seven resections, of which five were pathologically positive. In one patient with mucinous PM, the suspected lesion in the right inguinal canal turned out to be a fluid collection during surgery, and no resection was performed.

In PSS-3 patients, MRI identified lesions in the cardiophrenic space in one case, resulting in one resection that was pathological positivity. No lesions were detected in the inguinal orifice.

In total, across these two regions, preoperative MRI tagged 11 lesions, leading to 10 resections in 10 patients, of which 8 (80.0%) were positive in pathological examination.

## Discussion

In the cohort of patients with CRC-PM with surgical history and treated by CRS-HIPEC, preoperative MRI was used to tag lesions in seven areas of interest at risk of being overlooked or challenging to access due to prior dissection. While the diagnostic accuracy of MRI was unperfect, it demonstrated high specificity and moderate sensitivity and appeared particularly useful in patients with more extensive surgical histories. Importantly, the imaging strategy facilitated the detection of occult lesions in non-standard-regions such as the cardiophrenic space and inguinal canal.

The completeness of CRS is a major prognostic factor for patients with CRC-PM. Accordingly, accurate preoperative assessment of PM extension throughout the peritoneal cavity is necessary, not only for determining resectability, but also for enhancing the likelihood of complete resection, associated with improve survival.^27^ Computed tomography (CT) is commonly used to detect PM from CRC. However, CT tends to underestimate intraoperative PCI. Furthermore, CT sensitivity in detecting peritoneal implants is strongly influenced by lesion size, with a sensitivity of only 11% for small nodules (< 0.5 cm) compared with 94% for nodules > 5 cm.^17^ Staging laparoscopy continues to be the reference for assessing the peritoneal cavity. It ideally includes a slight extension of the open trocar to allow for palpation (of the small intestine particularly) in addition to observation.^28^ It enables the detection of radiologically occult PM, thereby optimizing resectability assessment and surgical planning elaboration. However, staging laparoscopy has drawbacks. Its utility can be significantly limited by adherences issued from prior surgical interventions. These adhesions may hinder complete exploration, leading to underestimation of PCI, or in some cases, cause serious injuries to abdominal organs.^29^ In PSS-2/3 patients, a staging laparoscopy prior to CRS may thus be impossible.^30^ Despite these limitations, staging laparoscopy remains crucial to determine resectability level, particularly regarding the small intestine attempt. Nevertheless, preoperative mapping of PM can still be improved to enhance the likelihood of achieving complete CRS. In this study, preoperative MRI was not intended to replace or challenge other imaging modalities or staging laparoscopy for resectability evaluation, but rather to serve as a complementary tool to guide surgical exploration and enhance PM detection.^31^ Laparoscopic exploration has inherent limitations and cannot adequately assess certain anatomical regions, including the abdominal wall, the inguinal canal, the cardiophrenic space, the posterior part of the porta hepatitis, and the retroperitoneal nodes. Several of these locations are associated with poor prognosis or increased risk of recurrence after CRS-HIPEC.^25,32,33^ In the present study, MRI demonstrated high specificity, reaching 98% for anastomosis regions. The MRI-guided strategy evaluated here appeared to enhance surgical exploration by identifying lesions that might have been missed during standard intraoperative assessment. While the benefit was difficult to demonstrate for the five areas of interest routinely explored during CRS, it was clearly evident in the ten lesions resected in ten patients in the cardiophrenic angle and inguinal canal areas. Thanks to that specific mapping, 80% were actually positive at pathologic analysis. These lesions were located in areas out of the scope of standard peritoneal cavity exploration. This approach contributed to improvement of CRS completeness, potentially leading to better prognosis.

Tumor cellularity is well explored with diffusion-weighted MRI.^34^ As most tumors exhibit high cellularity, even small tumor deposits (2–3 mm) typically light up against the dark nontumoral background signal.^18^ The soft tissue contrast of MRI makes it a valuable problem-solving tool for PM compared with CT.^5,35^ When combined with diffusion-weighted imaging—already known to provide a very high sensitivity (97%) in detecting liver metastases—the T2-weighted imaging and post-contrast sequences complement each other.^36,37^ In our study, MRI demonstrated moderate sensitivity (41–65%), high specificity (81–98%), and variable but relevant NPV (49–82%) for the regions of interest. Importantly, the rate of false positives was low, suggesting that when a lesion identified by MRI in a surgically challenging or high-risk area, intraoperative exploration should be strongly considered by the surgeon to achieve complete resection. Conversely, the sensitivity of peritoneal MRI for the regions of interest is moderate. Therefore, in the absence of visible PM on preoperative peritoneal MRI, the surgeon disposes of additional information to assess the benefit–risk balance of an iterative dissection at risk of morbidity.

Overall, the results showed excellent specificity for both PSS-2 and PSS-3 patients (81–98% and 83–100%, respectively) and good overall performance (63–81% and 64–86%, respectively), despite moderate sensitivity (38–64% and 50–73%, respectively). Notably, both PPV and NPV varied significantly by anatomical location. For instance, the perihepatic region and abdominal wall were associated with low PPV (77% and 60% in PSS-2 group, respectively) and high NPV (82% and 83% in PSS-2 group, respectively). Conversely, pelvis and anastomoses were associated with high PPV (82% and 93% in PSS-2 group, respectively) and low NPV (50% and 69% in PSS-2 group, respectively), primarily due to a higher rate of false negative in these regions. These discrepancies likely stem from postoperative changes in these areas, where distinguishing between postoperative fibrosis and recurrence can be difficult. Additionally, detecting lesions in these two regions, particularly those adhered to the serosa, can be challenging. Lesions may appear as plaques, nodules, or masses, but small, flat deposits can be missed, especially when they are closely adherent to the bowel or other organs. The complex nature of these lesions, their location near moving organs, and the presence of partial volume effects in MRI imaging contribute to these detection difficulties.^38^ Given these challenges, it is essential to interpret imaging in light of the patient’s previous examinations. Radiologists must be aware of the dates and nature of prior surgeries, as well as any surgical complications, to accurately differentiate between postoperative changes and recurrence.^39^

Unexpectedly, MRI performance was better in PSS-3 patients than in PSS-2 patients, despite comparable median PCI (median PCI = 6 [3–10] versus 5 [3–10], respectively). Two hypotheses may explain this finding. First, more extensive prior surgeries could have led to more adherences within the abdominal cavity, leaving less possibility for PM to spread. Second, surgical-induced anatomical changes in PSS-3 patients may have altered the radiologic appearance of the abdominal cavity, potentially enhancing the visibility of PM on MRI. However, these results should be interpreted with caution given the low number of patients (36) in the PSS-3 group.

This study has several limitations. First, its retrospective design, although based on a large patient cohort, introduces inherent biases. All peritoneal MRIs were interpreted by an experimented radiologist specialized in peritoneal surface malignancies. Yet, developing expertise in MRI for peritoneal disease remains particularly challenging, given the steep and prolonged learning curve.^44^ Accurate assessment of peritoneal involvement necessitates not only significant clinical experience, but also advanced technological tools. Therefore, improving imaging outcomes extends beyond enhancing diagnostic skills; it also requires technological advancements in MRI. MRI performance is further limited by motion artefacts from bowel peristalsis and susceptibility artefact on DWI.^40^ Recent deep-learning-based reconstruction algorithms have shown promising results that will overcome these limitations.^41–43^ Such advancements are essential to improving the precision of preoperative planning, potentially reducing the extent of unnecessary surgical dissections and ultimately improving postoperative outcomes. Another limitation was methodological; this study aimed at assessing the benefit of an imaging strategy within the context of a comprehensive, multimodal management approach for a complex disease: CRC-PM. The primary objective was to assess whether preoperative MRI could identify PM that might have been missed during surgical exploration. However, due to the retrospective design and the lack of comparison with routine CT evaluation, it was not possible to reliably determine all instances where MRI uniquely contributed to lesion detection, except for the inguinal canal and cardiophrenic areas. Furthermore, the surgical reports analysis did not allow for clear differentiation between preventive excision and truly suspicious retroperitoneal deposits. As a result, MRI performance was probably underestimated for this location.

To conclude, in a cohort of 248 patients with prior surgical dissection undergoing CRS-HIPEC for colorectal PM, preoperative peritoneal MRI demonstrated good specificity, interesting NPV, but modest sensitivity in 7 potential hidden regions. Preoperative MRI did not allow for exclusion of disease in these areas, but may have served as a useful adjunct to surgical planning by highlighting regions that warranted careful intraoperative exploration. Further studies are needed to clarify the added value of preoperative peritoneal MRI compared with standard preoperative imaging protocols.
